# Radiology in the era of value-based healthcare: a multi-society expert statement from the ACR, CAR, ESR, IS3R, RANZCR, and RSNA

**DOI:** 10.1186/s13244-020-00941-z

**Published:** 2020-12-21

**Authors:** Adrian P. Brady, Jaqueline A. Bello, Lorenzo E. Derchi, Michael Fuchsjäger, Stacy Goergen, Gabriel P. Krestin, Emil J. Y. Lee, David C. Levin, Josephine Pressacco, Vijay M. Rao, John Slavotinek, Jacob J. Visser, Richard E. A. Walker, James A. Brink

**Affiliations:** 1 Mercy University Hospital, Cork, Ireland; 2 Montefiore Medical Center, New York, USA; 3 University of Genoa, Genoa, Italy; 4 Medical University Graz, Graz, Austria; 5 Monash University, Melbourne, Australia; 6 Erasmus Medical Center, Rotterdam, The Netherlands; 7 Langley Memorial Hospital, Langley, Canada; 8 Thomas Jefferson University, Philadelphia, USA; 9 McGill University, Montreal, Canada; 10 Flinders Medical Centre and Flinders University, Adelaide, Australia; 11 University of Calgary, Calgary, Canada; 12 Harvard Medical School, Boston, USA; 13 European Society of Radiology (ESR), Vienna, Austria; 14 American College of Radiology (ACR), Reston, USA; 15 Royal Australian and New Zealand College of Radiologists (RANZCR), Sydney, Australia; 16 International Society for Strategic Studies in Radiology (IS3R), Vienna, Austria; 17 Canadian Association of Radiologists (CAR), Ottawa, Canada; 18 Radiological Society of North America (RSNA), Oak Brook, USA

**Keywords:** Radiology, Value, Value-based healthcare, Quality, Resources

## Abstract

**Background:**

The Value-Based Healthcare (VBH) concept is designed to improve individual healthcare outcomes without increasing expenditure, and is increasingly being used to determine resourcing of and reimbursement for medical services. Radiology is a major contributor to patient and societal healthcare at many levels. Despite this, some VBH models do not acknowledge radiology’s central role; this may have future negative consequences for resource allocation.

**Methods, findings and interpretation:**

This multi-society paper, representing the views of Radiology Societies in Europe, the USA, Canada, Australia, and New Zealand, describes the place of radiology in VBH models and the healthcare value contributions of radiology. Potential steps to objectify and quantify the value contributed by radiology to healthcare are outlined.

## Key points

- Value-based healthcare (VBH) is a framework for improving individual patient health outcomes per unit of expenditure.
- Radiology is a key component of healthcare, impacting greatly on patient outcomes, and must be considered a vital element of VBH.
- Embracing VBH principles, radiology can contribute to moving to a value-driven system, where all investigations or interventions contribute positively to patient outcomes.

## Introduction

In September 2020, members of this writing group published an article in JAMA on Radiology and Value-Based Healthcare [1], intended to raise awareness among non-radiologists of the value contributed to healthcare by radiology, and of ways that value can be harnessed and enhanced by those who utilise and those who deliver radiology services. This paper expands on that publication, in order to further explore the issues surrounding value-based healthcare as they involve radiology, and is primarily aimed at a radiology readership.

Value-based healthcare (VBH) has emerged in recent years as a framework for improving individual patient health outcomes per unit of expenditure [2, 3]. The impetus for this is, at least in part, the inexorable worldwide rise in healthcare usage volume and associated costs, increasing at a rate substantially greater than other cost-of-living inflation. The thrust of the VBH concept is to continue to improve individual health outcomes without commensurate increasing expenditure, by focusing on identification of practices that optimise the ratio between health gained and healthcare cost. The goal is to ensure that inflation does not make current healthcare systems unsustainable, while maintaining or continually improving patient outcomes.

US medical service funding is already influenced by traditional cost-effectiveness analyses (CEA) and the more recent VBH concept, as well as the related, but not necessarily aligned, value-based payment (VBP) models [4]. CEA focuses on a single metric (incremental cost effectiveness ratio, ICER) and is commonly used by policymakers to inform population—level decisions about which procedures, pharmaceuticals or devices will be funded or subsidised. “Value” in the context of VBH, on the other hand, focuses on what is of value to the individual during a particular episode of care and its immediate aftermath. Consequently, it remains less well-defined, with a wide range of proposed metrics. These patient-centred metrics are, in turn, not necessarily aligned with VBPs (e.g. US Medicare and Medicaid Value-Based Payment Modifier), which often focus on short-term costs to a specific payer of an episode of care. Criticisms of such systems revolve around their inability to accurately measure important patient outcomes and their potential to exacerbate existing disparities in care delivery without improving physician performance of healthcare delivery [5].

The European Commission Expert Panel on Effective Ways of Investing in Health has recently published a draft Opinion Paper on “Defining Value in ‘value-based healthcare’”, which seeks to move the discussion away from value-based pricing to a broader definition of VBH, based on four pillars:

- appropriate care to achieve patients’ personal goals (personal value)
- achievement of best possible outcomes with available resources (technical value)
- equitable resource distribution across all patient groups (allocative value)
- contribution of healthcare to social participation and connectedness (societal value) [6]

Whatever the source of funding in any individual country, it is likely that healthcare institutions will be obliged in the future to demonstrate that they apply VBH principles and optimise resource utilisation in order to ensure continued funding. Therefore, not only is VBH a sensible approach to guide critical assessment of practices; it also will be key to services maintaining future financial viability.

This paper, written by representatives of the European Society of Radiology (ESR), American College of Radiology (ACR), Radiological Society of North America (RSNA), Canadian Association of Radiologists (CAR), Royal Australian and New Zealand College of Radiologists (RANZCR) and International Society for Strategic Studies in Radiology (IS3R), seeks to outline the value contributed to healthcare by radiology, and to explain how that value may be measured, recognised and augmented.

## Value-based healthcare models

Porter’s original description of a VBH model listed an outcome measure hierarchy containing three tiers (Sustainability of Health, Process of Recovery & Health Status achieved or retained), with many factors contributing to each tier. The top tier (Sustainability) is considered the most important, with lower-tier outcomes involving results contingent on higher-tier success [8]. In his 2010 NEJM paper outlining this model, Porter acknowledged that medical care “involves multiple medical specialties and numerous interventions”, and that “[m]uch of the total cost of caring for a patient involves shared resources, such as physicians, staff, facilities, and equipment” [7]. When calculations of value are used as a basis for resourcing or reward, conflicts can develop between different groups of contributors to care [1]. Porter writes: “in a well-functioning health care system, the creation of value for patients should determine the rewards for all other actors in the system” [7].

Radiology is a vital part of modern medicine, a significant positive contributor to patient diagnosis and continuing care, and thus a key component of provision of value. Furthermore, radiology as a specialty is the perfect example of a healthcare resource shared across all levels of healthcare delivery, all medical specialties, and patient care at all ages [1]. Diagnostic radiology contributes value in clinical workup by refining differential diagnoses formulated from history-taking, physical examination and sometimes laboratory test results, thereby decreasing the time required to initiate appropriate treatment, ultimately helping to reduce patient morbidity and mortality [8, 9]. In Porter’s VBH model, health gains and reduced costs associated with decreased time in hospital, improved survival and lower utilisation of ineffective treatments and investigations are not recognised as contributions made by radiology to the value of healthcare. Nonetheless, short-term expenditures on imaging may create long-term and system-wide cost savings and better patient outcomes, none of which are credited to the value of radiology according to this model.

One extreme interpretation of the VBH model considers diagnostic radiology as a “cost centre”, whereby all expenditures on imaging are perceived to negatively contribute to value in healthcare, in the context of the influence of errors or complications negatively affecting outcomes in the Process of Recovery tier. Errors happen in radiology, as they do in all branches of medicine, but many reports of errors in radiology misunderstand the diagnostic process, and apply biases to interpretation after the fact, rather than reflecting the reality of interpretation of imaging data at a specific time, often based on limited background information [3, 10]. This extreme view values radiology’s contributions (if at all) in much the same way as laboratory investigation outputs, ignoring much of the value created by the practice of radiology, and radiology’s clinical centrality to patient care.

## Radiology’s place in value-based healthcare models

How, then, can we ensure that radiology is appreciated not as a potential source of loss of value, but rather as an intrinsic value creator?

**Fig. 1:**
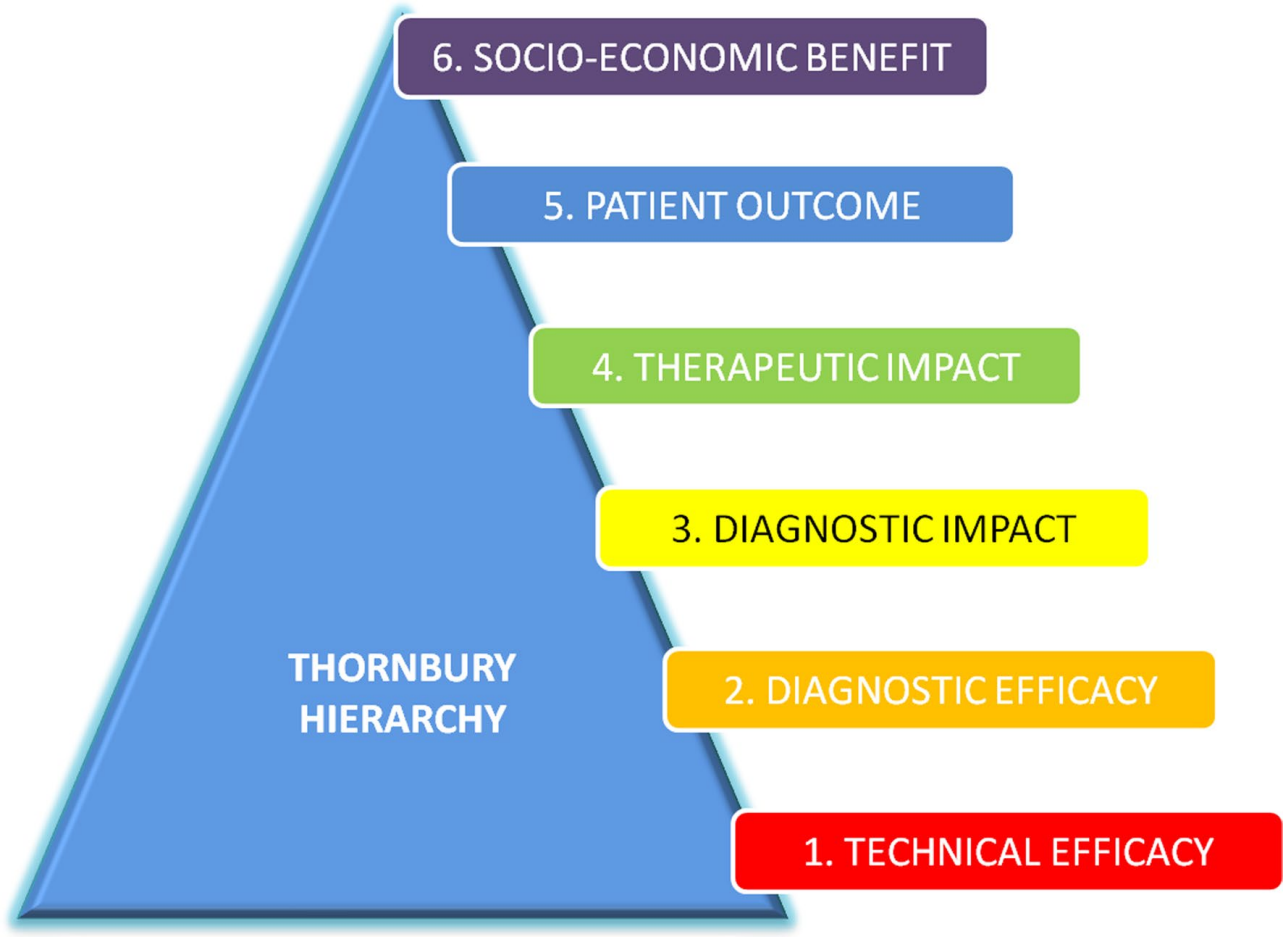
Hierarchical value model. (Reproduced with permission from Raja UA, Patel S, Singh LK, Shah D, Hamdulay S, Penn H, Remedios D. Early arthritis ultrasound: a 4-year outcome study. ECR 2014, EPOS, 10.1594/ecr2014/C-2059)

The most important way to do this is to quantify radiology’s impact on patient outcomes and on measurements used historically by policymakers and other third party payers, such as Quality Adjusted Life Years (QALYs) and ICERs. In 1991 Fryback and Thornbury proposed a 6-level hierarchical value model starting with evidence of technical efficacy at the lowest level and ending with societal efficacy at the highest level [11] (Fig. 1). It is generally considered that adding value to patient care only starts at level 4. However, much scientific literature relating to diagnostic radiology (as opposed to image-guided therapy) relates to image acquisition and diagnostic accuracy, at levels 1 and 2, rather than to the contribution of radiology, in concert with the entire system of delivery of care, to the health outcomes of the patient or society as a whole (the higher hierarchical levels). For instance, a high quality MRI performed on well—maintained equipment by a highly trained radiologist for a previously well 42—year old patient reporting two weeks of non-specific low back pain (effective at levels 1 & 2) may provide less net benefit to individual or societal health than an average quality head CT for a 25 year old painter who fell from a ladder and has a high pre-test clinical risk of intracranial injury (effective at levels 3–6) [11].

Diagnostic Radiology faces special challenges in demonstrating a link between its key output, (making or changing a diagnosis), and the final step in the value chain, (improved health of the patient), due to the many confounders along the pathway between diagnosis and outcome.

Pathways exist for radiology providers to demonstrate meaningful contributions to patient health outcomes, or to have their funding/reimbursement influenced by value-based activity. The US Medicare Access and CHIP Reauthorization Act of 2015 (MACRA) established the Quality Payment Program, under which eligible clinicians can participate via one of two tracks: Advanced Alternative Payment Models (APMs); or the Merit-based Incentive Payment System (MIPS). Both tracks involve quality measures that demonstrate participation in certain quality improvement activities as well as contributions of radiology activity to patient care [12].

Considering the issues underpinning radiology value is not a new idea. In 2009, Gunderman & Boland elegantly outlined some of the reasons physicians or patients might choose to use one radiology service over another (perceived relative value), and some of the questions radiologists might ask themselves when considering the value they provide to patients [13]. In 2011, Rao & Levin explained the value-based benefits to patients of single, cohesive, on-site radiology groups in hospitals, as opposed to fragmented or out-sourced imaging services [14]. Also in 2011, Gazelle et al. [15] proposed a framework to assess the value of diagnostic imaging in the era of comparative effectiveness research. In 2016 Seidel et al. [16] described specific strategies for diagnostic imaging to generate evidence and value.

Nobody in modern medical practice could imagine attempting to function and maintain standards of clinical service in the absence of diagnostic imaging services, including specialist radiologist interpretation, consultation and intervention. Radiology is a deeply-embedded and essential part of modern patient care, at all levels of service delivery and complexity, encompassing high-level hospital-based medicine, primary care investigation, screening and health-promotion activities. “Few episodes of care occur without medical imaging, and a rational health care system should define the distribution of revenue to radiology based on its value as derived from quality and costs” [17].

Radiology departments have the potential to be bottlenecks in any healthcare environment. A secondary analysis of the US National Hospital Ambulatory Medical Care Survey (NHAMCS) from 2006 through 2008 demonstrated that when a physician ordered an ultrasound, CT, or MRI during an emergency department visit, the average length of stay for that patient increased by 56, 59, and 64 min respectively [18]. Under-resourced hospital-based services can delay patient throughput and discharge. Under-resourced primary-care and out-patient access to imaging limits the capacity of non-hospital-based services to manage patients, resulting in increased reliance on more expensive hospital-based facilities. Adequate resourcing of radiology is vital to achieving or maintaining healthcare efficiency, and thus to maximising value. Therefore, as part of the fundamental goal of enhancement of value for patients, radiology must be a component of any formula to assess costs against outcomes in healthcare.

## Value equation

Relating technical quality, service quality and price has been defined as the radiology “value equation” [19]. What constitutes value in healthcare depends upon who you ask. The University of Utah Health surveyed patients, physicians and employers who pay for medical benefits, in an effort to define how they perceive value. Each group prioritised different value statements, reflecting the different viewpoints of those who deliver a service, those who receive it and those who pay for it [20]. This led the authors to propose a shift from the original Porter equation (Value = Outcome/Cost) to a more-nuanced one, identifying service as a specific component (Value = Quality + Service/Cost). Quality may incorporate elements such as employee productivity (for employers) that matter little to other groups. Service may include elements such as out-of-pocket expenses (for patients) that are not prime considerations for physicians or employers.

Value exists as a concept only in the eyes of the recipient. In economic terms, it can be considered as the total amount of money a customer would be willing to pay for a service. Value creation involves providing new services or improving existing services to increase their worth to the recipient, at little or no additional cost [19].

## Where is the value of radiology delivered?

- Prevention
  - Disease prevention (screening and predictive imaging biomarkers)
  - Reassurance, e.g. confirmation of the absence of disease, eliminating the need for further (potentially-expensive) investigation
  - Radiation protection – optimising protocols to minimise risk, preventing unnecessary or duplicate studies.
- Detection
  - Population-based screening programs
  - Identification of abnormalities accounting for clinical presentations
- Diagnosis
  - Disease staging, facilitating decisions about appropriate management
  - Provision of high-level subspecialist imaging interpretation, shown to improve staging and management decision-making [21]
  - Image-guided lesion biopsy for histopathology
  - Clinical decision-support – facilitating the choice of the most-helpful and most-targeted investigation to answer a clinical question and indicating clinical situations in which imaging is likely to represent low-value care.
- Delivery and monitoring of therapy
  - Evaluation of patient progress during treatment; early treatment monitoring (responders vs. non-responders)
  - Development & utilisation of imaging biomarkers, to facilitate earlier disease detection, prediction of response to treatment, reduction in invasive testing and improvements in targeted treatments. Imaging biomarkers add value to pre-treatment workup, treatment choice and follow-up for many conditions. Biomarkers can act as surrogate endpoints in clinical trials, leading to more rapid translation of research to clinical practice [22].
  - Interventional radiology – minimally-invasive investigations and treatments, often resulting in speedier recovery than after formal surgery
- Prognosis
  - Confirmation of disease resolution, facilitating cessation of treatment
- Other
  - Teleradiology linking rural communities and highly specialised radiology centres/hospitals
  - Other non-interpretive activities, e.g. teaching, multi-disciplinary team meeting preparation and participation, research and administrative work [23]
  - Communication to patients, the public, the medical community and other interested stakeholders. This includes critical test result notifications to ensure timely clinical handover and emergency care [1].

## How is value measured?

Impacting therapeutic decisions, improving patient outcomes and benefits for society as a whole are the core aspects of value creation in radiology. Quantifying radiology’s impact requires more precise, reproducible, and practically-measurable imaging-specific and clinically-relevant metrics linked to agreed and important health outcomes. Future radiology research must place greater emphasis on Fryback and Thornbury’s higher-level outcomes [11] to best demonstrate radiology’s value. While a diagnostic test such as breast MRI, performed using the same equipment, scanning parameters, and interpreter, may have equivalent diagnostic performance in two different patient groups, its efficacy will likely be greater in women with specific characteristics (e.g. BRCA1 mutation carriage).

## To whom is the value of radiology delivered?

Ultimately, the recipient of healthcare services (and value) is the patient, and, to some extent, their loved ones. However, except in the context of screening, requests for diagnostic radiology studies usually come from referring clinicians who seek radiology’s input, and directly receive the output (reports). Referring clinicians can be considered as “intermediate customers”. When optimally utilised, the value of radiology is also delivered to hospitals and health services and to the economy as a whole [1].

Patients do not want an ultrasound, CT, or MRI; they want an answer to a clinical question. The primary purpose of diagnostic radiology is to answer clinical questions using medical imaging, and to help guide patient care in the most effective way possible, including in some instances not performing an imaging test [1]. Fundamentally, diagnostic radiology is concerned with acquisition, utilisation, and dissemination of information [1]. Process metrics can be used to measure aspects of value delivery including timeliness of information delivery, application of appropriate levels of specialisation to interpretation (and thus to accuracy of information acquisition), and tailoring of information delivery to the needs of different types of intermediate customers (e.g. emergency care, primary care, non-urgent specialty care) [19].

## What is the goal?

Radiology is a key component of healthcare, impacting greatly on patient outcomes, and must be considered a vital element of VBH. Radiology must be part of any calculation of value metrics, and resourcing decisions based on such calculations must take account of the need to resource radiology adequately to maintain its value contribution [15].

Radiologists and radiology departments have a responsibility to help define and create value wherever possible and to optimise the yield from what we do. In addition, we need to publish research reporting on radiology’s impact on therapeutic decisions, patient outcomes, and societal benefits, especially when targeting select patient populations for new medical imaging applications, when associated healthcare costs may be large [15]. Traditional radiology research metrics like diagnostic and technical accuracy may be sufficient to demonstrate a value contribution for tests and procedures with smaller, well-defined target populations and/or clear impacts on patient outcome [15]. When assessing the societal value of radiology, we need different robust, reproducible, and clinically relevant outcome metrics to objectify and quantify the value contributed by radiology [24].

Steps which can support this endeavour include:

1. Engaging directly and often with referring clinicians to better understand their practices and needs, and to develop mutual relationships of trust and understanding.
  - Supporting evidence-based guidelines to assist referrers in requesting appropriate imaging or interventional procedures specific to the patient’s clinical history or condition (e.g., ESR iGuide, ACR Appropriateness Criteria, Choosing Wisely) [25–28].
  - Reinforcing the use of such guidelines in collaboration with referrers enhances the quality of patient care and enables radiologists to contribute value through efficacious resource use.
2. Understanding the varying needs of referrers (e.g. rapid turnaround, 24/7 availability for emergency care, subspecialty expertise, multidisciplinary input for complex, non-emergency cases), and building services to encompass all needs without conflict [1].
3. Ensuring that radiology departments work cohesively as a whole, operating as teams to ensure enterprise-wide standards are achieved, cross-cover and -support are freely available, and isolated silos do not develop to the detriment of other areas of service.
4. Structuring department work plans to meet referrers’ needs, e.g. making protected time available for multidisciplinary team activity.
5. Utilising available resources and tools (e.g. structured reporting, clinical decision support tools, AI tools) and, where possible, augmenting resources to optimise workflow to minimise patient waiting times for studies, and (if achievable) shorten hospital stays
6. Engaging directly with patients, to answer their questions and offer explanation of their imaging findings, as appropriate [1].
7. Optimising information (images, reports etc.) exchange using appropriate IT tools, e.g. provision of urgent report notifications, clinical decision support tools and use of structured reporting, including links to key images demonstrating positive findings [29].
8. Constant quality monitoring and promotion of a culture of constant quality improvement [19].
9. Experimental research, including efforts to establish higher-level value contributions: supporting today’s radiology research is a commitment to improving tomorrow’s radiology practice [30].

These principles are inherent to several value-based imaging initiatives, including the ACR’s Imaging 3.0 [31], the RSNA’s Radiology Cares [32], and the RANZCR’s Inside Radiology [33]. Optimisation of value-creation and resource utilisation demands cooperation among all those involved, including referrers, patients, healthcare administrators, and radiologists. Patients must understand that their specific needs are best served by a flexible, responsive healthcare system that applies the investigation best suited to answering the relevant clinical question at that particular point in their care, with the greatest safety. Referrers must work with radiologists to optimise resource utilisation, justified and optimised to the specific patient’s circumstance at the time, in order to maximise value. All parties must educate themselves about methodologies used to determine costs and value, and must understand that their choice of actions and decisions may have influences that go far beyond the narrow specifics of any one episode of patient care or siloed departmental or hospital budgets. Cost calculation and allocation is complex and relative, depending on the reference points used [17].

## Conclusion

VBH as a concept is here to stay. It will underpin future planning and resource allocation in all aspects of medical care. Models of defining value remain in evolution. Narrow models which commence the consideration of value with the making of a diagnosis are incomplete, and misrepresent the entire healthcare resource allocation for that patient. Radiology’s contribution to healthcare is broad, encompassing many aspects that go beyond traditional study report creation. Objectifying this contribution by stating the impact on therapeutic decisions, patient outcomes, and societal benefits ensures radiologists’ future role. Radiologists, working singly or as parts of collective departments, must understand the principles underpinning cost allocation and the value-chain concept, and must take VBH into account when planning, developing and delivering their services. Equally, referrers, who impose costs without incurring them directly (by utilising services which are paid for by patients or third party payers) must have greater accountability for their impact on the cost of medical imaging and for ensuring resources are utilised for optimum patient health benefit. Managers who resource and plan healthcare services must understand how under-resourcing of potential bottlenecks in service delivery, such as radiology facilities, can impact negatively on outcomes for patients. By embracing VBH principles, and striving to create value where possible, radiology can contribute greatly to moving from a volume-driven system to a value-driven one, where as many investigations or interventions as possible contribute positively to patient outcomes [1]. This will require renewed willingness on the part of radiologists to participate in team-based clinical decision-making with other specialists. It will also require willingness on the part of referrers to work with radiologists to ensure the most appropriate use of radiology resources, services and personnel [1].
